# Gross on the Urinary Organs

**Published:** 1877-05

**Authors:** 


*A Practical Treatise on the Diseases, Injuries, and Malformations of the Urinary Bladder, the Prostate Glands, and the Urethra.* By SAMUEL D. GROSS, M. D., LL.D., D. C. L. Oxon., etc, etc. Third Edition, Rivised and Edited by Samuel W. Gross, A. M., M. D. Illustrated by One Hundred and Seventy Engravings. Philadelphia: Henry C. Lea, 1876.

The former editions of this work have been well known to American readers, it was indeed until the appearance of the excellent work of Van Buren and Keyes, the only American work of any importance upon the subject. In its present form it comes to the reader as almost a new book, so many are the changes which have been made in its general character.

The present edition, edited by Dr. S. W. Gross, has in many of its points been quite materially changed. The chapters upon the Anatomy of the Urinary Organs, and an appendix upon the prevalence of stone in the bladder and calculous disorders in the United States have been omitted from this edition. The present Editor has introduced chapters upon "Tumors of the Bladder" and upon the Prostate Gland" which are entirely new. Several other chapters contain a large amount of new material, notably those upon Stricture, Lithotomy, and Diseases of the Bladder. On page 290 in speaking of median lithotomy the author says:—"the operation is only adapted to small calculi. That this last objection is a most serious one is shown by the analysis of Mr. C. Williams of sixty-four cases of median lithotomy at the Norfolk and Norwich Hospitals. The entire number of deaths was thirteen; and in no instance did recovery result when the stone weighed over three drachms and two scruples, except in the case of a man, forty years of age, in which the concretion exceeded four ounces and a half, but it was followed by sloughing of the rectum and perineum, and the establishment of a permanent perineo-recto-vesical fistula."

From what we have seen of this operation, it does not seem adapted to the extraction of large stones in adults, and we can readily see the force of the addition which follows the above quotation, that death occurred in cases over fifty-two. It is to the extraction of stone in children that this method seems very excellently adapted. The membranous urethra is capable of a large amount of dilatation, and the prostate is so small as to practically be out of the way. In a case reported in this Journal (Vol. XIV, page 324,) the calculus weighed 706 grains, and measured (over the forceps) 1⅝ inches in thickness by 1⅛ inches (between the blades) in breadth. The patient, a lad aged twelve, made a good recovery.

There is very little however in this work to criticise, much to admire. The illustrations are excellent, and some of them, taken from preparations in the Army Medical Museum, illustrate the value of the work there accomplished. The author and editor are to be congratulated upon the production of the present edition, which all points is an excellent presentation of the subject.

